# miR-33 and RIP140 participate in LPS-induced acute lung injury

**DOI:** 10.3906/sag-1804-173

**Published:** 2019-02-11

**Authors:** Hua LI, Huan HOU, Shuang LIU, Yangyang FENG, Wansi ZHONG, Xiaojuan HU, Nianlong YAN

**Affiliations:** 1 Department of Biochemistry and Molecular Biology, School of Basic Medical Sciences,Nanchang University, Nanchang, Jiangxi P.R. China; 2 Basic Medical Experiments Center, Nanchang University, Nanchang, Jiangxi P.R. China

**Keywords:** Lipopolysaccharide, PMVEC, acute lung injury, miR-33 and RIP140

## Abstract

**Background/aim:**

Pulmonary microvascular endothelial cells (PMVECs) play a pivotal role in the process of acute lung injury (ALI), which can be induced by lipopolysaccharide (LPS). Numerous reports have indicated that both miR-33 and RIP140 are involved in the inflammatory response in macrophages. In this study, we sought to investigate whether miR-33 and RIP140 participate in ALI induced by LPS.

**Materials and methods:**

First, we isolated and identified PMVECs from BALB/c mice. Subsequently, both PMVECs and BALB/c mice were treated with PBS, LPS, or pyrrolidine dithiocarbamate (PDTC) plus LPS and divided into three groups: control (PBS), LPS (LPS), and L+P (LPS plus PDTC) groups. We assessed pathology by hematoxylin and eosin staining, and miR-33 and RIP140 expression levels were examined using quantitative PCR and Western blot analyses.

**Results:**

Our results demonstrated that LPS can induce PMVEC injury and ALI and that LPS treatment significantly decreased miR-33 expression compared with controls (P < 0.001, n = 5). On the contrary, RIP140 was markedly overexpressed by LPS treatment (P < 0.001, n = 5). However, this alteration can be inhibited by pretreatment with PDTC before LPS (P < 0.05, n = 5).

**Conclusion:**

This study is the first to confirm that both miR-33 and RIP140 participate in LPS-induced PMVEC injury and ALI, which may help uncover the mechanism of ALI.

## 1. Introduction

Acute lung injury (ALI) is a critical clinical disease caused by uncontrolled inflammation (1). Mortality due to ALI is high at 35%–40%, but its detailed pathogenic mechanism remains unclear. Overexpression of inflammatory mediators as a result of a variety of factors excluding cardiogenic factors can lead to ALI, and sepsis represents one of the major factors (2). Lipopolysaccharide (LPS) is the main component of the outer membrane of gram-negative bacteria, which are a major pathogenic cause of sepsis (3). In sepsis, LPS can attack pulmonary microvascular endothelial cells (PMVECs), resulting in leakage of protein-rich edema fluid as a result of PMVEC injury and barrier dysfunction. Moreover, the main pathologic feature of ALI is pulmonary edema due to the increase in PMVEC permeability (4,5). Therefore, PMVEC function is receiving increasing attention in ALI research.

MicroRNA-33 (miR-33) is an intronic miRNA that encodes sterol-regulatory element-binding factor-2, which is involved in atherosclerosis, as it is the target of ATP-binding cassette transporter A1 (ABCA1) and ATP-binding cassette transporter G1 (ABCG1) (6,7). However, a recent study suggested that miR-33 also participates in inflammatory reactions. For example, when BALB/c mice were intraperitoneally injected with LPS to induce acute inflammation and ALI, miRNA microarray analyses revealed that LPS-induced inflammation significantly downregulates miR-33 expression by approximately 0.57-fold after 24 h (8). Furthermore, Jin et al. discovered that LPS and IFN-gamma stimulation can also significantly downregulate the expression of miR-33 in dendritic cells by approximately 1.41-fold (9).

Receptor-interacting protein 140 (RIP140) is a transcriptional coregulator of numerous transcription factors and a regulator of signal transduction (10–12). Previous studies have suggested that RIP140 can negatively regulate energy u2912 by affecting the storage of lipids and inhibiting the expression of genes involved in fatty acid oxidation and glucose metabolism (13). However, Zschiedrich et al. reported that genetic factors and acute deficiency of RIP140 lead to inhibition of the proinflammatory program in macrophages (14). When macrophages were treated with modified low-density lipoprotein (oX-LDL, an inflammatory stimulus), IL-1β and TNF-α levels were increased due to NF-κB coactivation by RIP140 (14). The mechanism involved the modification of low-density lipoprotein, which leads to the accumulation of cholesterol and activates the cholesterol-miR-33-RIP140 regulatory pathway that modulates the proinflammatory potential of macrophages in response to altered intracellular cholesterol status (10). Moreover, RIP140 overexpression can increase proinflammatory gene expression and cytokine release, and these effects can be reversed by p65-NF-κB inhibition in neonatal rat cardiomyocytes (15).

Clearly, both miR-33 and RIP140 are associated with inflammatory reactions. However, Guo et al. demonstrated, only by miRNA microarray analyses, that miR-33 expression was reduced; further validation by quantitative PCR analysis was not performed (8). Moreover, RIP140 is mainly expressed in metabolic tissues; thus, it remains unclear whether lung cells express RIP140 and its role in ALI. In addition, the possible relationship between RIP140 and PMVEC injury during inflammation remains uncharacterized. Therefore, to uncover their possible roles and relationship with miR-33 and RIP140 in LPS-induced ALI, both primary PMVECs and BALB/c mice were treated with phosphate-buffered saline (PBS), LPS, or LPS plus pyrrolidine dithiocarbamate (PDTC), which is a specific inhibitor of NF-κB (16,17). Subsequently, we assessed their pathology and detected miR-33 and RIP140 expression.

## 2. Materials and methods

### 2.1. Isolation and culture of mouse PMVECs

PMVECs were isolated from mouse lung tissue as previously reported (18). Briefly, three 6- to 8-day-old BALB/c mice were anesthetized. Then, strips of peripheral lung were removed, and the tissue was finely minced using sterile scissors. The tissue was incubated in 1% gelatin-coated 25-cm2 tissue culture flasks in growth media. All tissue was removed after 65 h. After subculture, PMVECs were seeded on slides and identified by immunofluorescence staining for factor VIII (BIOSS Biological technology, Inc., Beijing, China) expression. Slides were analyzed by inverted fluorescence microscopy.

### 2.2. Cell model of PMVEC injury

PMVECs were seeded in 6-well plates. Before treatment with 100 ng/mL LPS (Sigma‑Aldrich; Merck KGaA, Darmstadt, Germany), the L+P (LPS plus PDTC) group was treated with 100 μmol/L PDTC (Beyotime Institute of Biotechnology, Haimen, China). After 1 h, the LPS and L+P groups were exposed to LPS (100 ng/mL), while the control (PBS) group was treated only with PBS. Twelve hours later, the culture supernatant and cells were collected. The main pathologic feature of ALI is pulmonary edema due to increased PMVEC permeability. Moreover, when PMVECs were treated with LPS, their permeability changed, and LDH was released from PMVECs. Therefore, LDH is considered a major index of permeability levels (19), and LDH was measured using a Lactate dehydrogenase assay kit (Jiancheng Bioengineering Institute, Nanjing, China) in this study. The expression levels of miR-33 and RIP140 in cells were also measured.

### 2.3. Animal model of ALI

Fifteen BALB/c mice weighing 26 ± 3 g (5 to 6 weeks old) were obtained from the Experimental Animal Center of Nanchang University (Nanchang, China). All mice were maintained under a 12-h light/dark cycle and supplied with standard food and water. Sepsis was induced in mice as previously described (20,21). Briefly, the mice were divided into three groups (5 per group): control, LPS, and L+P (LPS + PDTC) groups. First, the L+P group was intraperitoneally injected with 30 mg/kg PDTC (PDTC was dissolved in 50 μL of PBS). Simultaneously, both the control and LPS groups were intraperitoneally injected with the same volume of PBS (50 μL). One hour later, mice in the LPS and L + P groups were intraperitoneally injected with 10 mg/kg LPS (LPS was dissolved in 50 μL of PBS), which was purchased from Solarbio (Beijing Solarbio Science & Technology Co., Ltd., Beijing, China). However, the mice in the control group were intraperitoneally injected with only 50 μL of PBS. Mouse survival was monitored every hour for 24 h. The mice were then euthanized by cervical dislocation, and the lungs were collected for analysis. This study obtained ethical approval from the Committee on Animal Experimentation of Nanchang University, and the procedures complied with the NIH Guide for the Care and Use of Laboratory Animals.

### 2.4. Hematoxylin and eosin staining

To ensure successful construction of the animal model of ALI, mouse lungs were fixed in 10% formaldehyde. The fixed tissues were then embedded in paraffin and cut into 4-μm sections using a microtome (Leica, Nussloch, Germany). The sections were placed on glass slides, deparaffinized, and stained with hematoxylin and eosin (21,22). These results were viewed using an inverted fluorescence microscope (IX-71 OLYMPUS).

### 2.5. Quantitative PCR analysis

RNA was isolated using RNAiso Plus reagent (Takara Biotechnology Co., Ltd., Dalian, China) according to the manufacturer’s protocol. Total RNA (0.5 μg) was used for reverse transcription in a total volume of 5 μL (Thermo Fisher Scientific, Inc., Waltham, MA, USA). RIP140, miR-33, β-actin, and U6 primers were synthesized by Invitrogen. The primer sequences were as follows: RIP140 forward GGCAGCAAACCTGAATTCGGC, reverse CTCACCGGGCACGGAACATC; β-actin forward TGAGCTGCGTTTTACACCCT, reverse GCCTTCACCGTTCCAGTTTT; miR-33 forward ACACTCCAGCTGGGGTGCATTGTAGTT, reverse CTCAACTGGTGTCGTGGAGT; and U6 forward GTGCTCGCTTCGGCAGCA, reverse CAAAATATGGAACGCTTC.

To measure the expression of these genes, SYBR Green was used as a fluorescent dye (Thermo Fisher Scientific, Inc.). The PCR program (ABI 7500 sequence detection system) comprised of 95 °C for 3 min and 40 cycles of 95 °C for 10 s and 60 °C for 1 min. Fluorescence data and melting curves were obtained. Experimental data represent the average and standard deviation of three biological replicates. RIP140 expression was normalized to β-actin expression, and miR-33 expression was normalized to U6 snRNA expression (23).

### 2.6. Western blot analysis

Proteins were extracted using radioimmunoprecipitation buffer (Kangwei Century Biotechnology Co., Ltd.), and protein concentrations were measured using a bicinchoninic acid (BCA) assay (Kangwei Century Biotechnology Co., Ltd., Beijing, China). Equal amounts of clear lysates were separated by sodium dodecyl sulfate-polyacrylamide gel electrophoresis (SDS-PAGE) and then transferred onto polyvinylidene fluoride membranes (Immobilon P, Millipore, Bedford, MA, USA). Equal transfer was validated by staining with Ponceau red. The membranes were blocked with 10% skim milk in Tris-buffered saline (TBS) and then incubated with primary antibodies in TBS containing 0.05% Tween 20, 2% bovine serum albumin, and 0.05% sodium azide overnight at 4 °C. The following antibodies were used at the indicated dilutions: RIP140 (Santa Cruz Biotechnology, Inc., Dallas, TX, USA) at 1:1,000 and β-actin at 1:10,000 (ProteinTech Group, Inc., Wuhan, China). Secondary horseradish peroxidase-conjugated antibodies (rabbit, ProteinTech Group, Inc., Wuhan, China) were used at 1:10,000 in 10% skim milk in TBS containing 0.05% Tween 20 (24). Signals were revealed using an enhanced chemiluminescence reagent (Kangwei Century Biotechnology Co., Ltd.) and an autoradiography system (CLINX, Chemiluminescence Imaging System).

### 2.7. Statistical analysis

Statistical analyses were performed using the statistical software SPSS22.0, and significant differences between groups were evaluated using Student’s paired t-test. For multiple comparisons, one-way ANOVA with Tukey’s or Games–Howell post hoc analysis was used. All experiments were repeated at least 3 times, and P < 0.05 was considered statistically significant.

## 3. Results

### 3.1. Identification of PMVECs and the cell injury model

**Figure 1:**
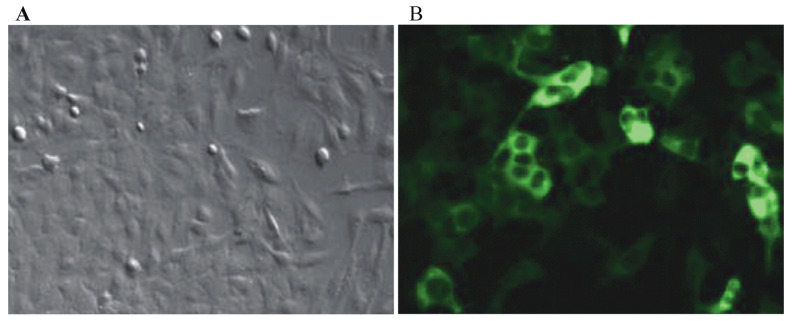
Identification of PMVECs. (A)-Inverted microscopy revealing PMVEC morphology. (B)-Identification of PMVECs by immunofluorescence staining for factor VIII expression.

**Figure 2:**
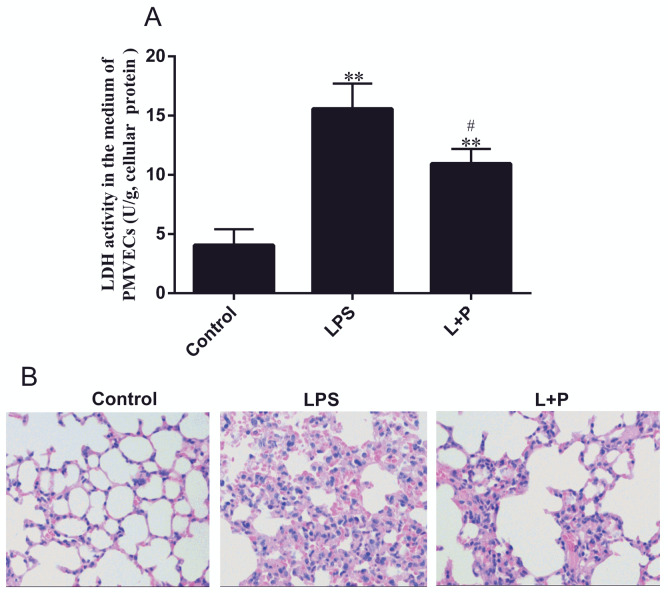
Evaluation of LPS-induced PMVECs and mouse lung injury. (A)-LDH levels in the PMVEC culture supernatant (μmol/g) are reported as LDH/protein. Values are presented as the mean ± SD (PMVEC n = 3; mouse n = 5). ** P < 0.001, the LPS group compared with the control group; # P < 0.05, the L+P group compared with the LPS group. (B)-Representative light micrographs of HE-stained lung tissues from the control, LPS, and L+P groups after 24 h.

To understand the possible function of PMVECs in ALI, we isolated PMVECs and constructed a cell injury model. Immunofluorescence staining results showed that the cells isolated from the mouse lung were true PMVECs, as shown by positive factor VIII expression (Figure 1). When cells were treated with 100 ng/mL LPS, the LDH levels in the culture supernatant were increased by approximately 3.16 ± 0.45-fold in the LPS group compared with those in the control group (Figure 2A, P < 0.001, n = 3). Compared with LPS, PDTC (L+P) significantly inhibited this change (P < 0.05, n = 3). However, the levels of LDH were increased by approximately 1.85 ± 0.36-fold (P < 0.001, n = 3) compared with those in the control group.

### 3.2. Identification of the ALI mouse model

Inflammation alters miR-33 and RIP140 expressions in PMVECs. Furthermore, we assessed miR-33 and RIP140 expression in the lungs of the ALI model mice. Therefore, we first constructed the ALI mouse model using LPS. To assess the pathological changes, HE staining was used in our study. As noted in Figure 2B, lung tissues from the control group exhibited normal structures. In contrast, exudate and blood were noted in the alveoli of the model (LPS) group, and alveolar wall capillaries were dilated and congested. In addition, dense lymphocyte infiltration was noted on the capillary wall (Figure 2B). However, the degree of injury in the L+P group was more severe than that in the control group and was less severe than that in the LPS group. In addition, exudate and blood in mouse lung tissue were not notable in the PDTC group, and minimal inflammatory cell infiltration was noted (Figure 2B). Hence, the mouse ALI model was successfully generated.

### 3.3. Expression of miR-33 in PMVECs and mouse lungs

**Figure 3:**
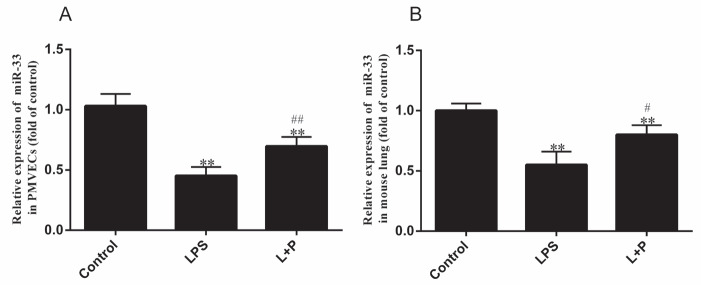
Expression of miR-33 in PMVECs and mouse lungs treated with LPS. The expression of miR-33 in PMVECs (A) and mouse lungs (B). Expression levels were analyzed by real-time quantitative PCR. Values are shown as the mean±SD (PMVEC n=3; mouse n=5). *P < 0.05 and **P < 0.001, the LPS group compared with the control group; #P < 0.05, the L+P group compared with the LPS group.

miR-33 is involved in atherosclerosis, which is a chronic inflammatory process. Thus, we measured miR-33 expression in injured PMVECs and mouse lung tissue induced by LPS. As demonstrated in Figure 3A, miR-33 expression was reduced by approximately 0.55 ± 0.06-fold in PMVECs (P < 0.001, n = 3). Moreover, Figure 3B shows that miR-33 expression was downregulated in the mouse lung after exposure to LPS. miR-33 expression levels were decreased by approximately 0.44 ± 0.11-fold compared with those in the control group (P < 0.001, n = 5). However, PDTC reversed this alteration (Figure 3) in PMVECs and mouse lungs. Compared with the control group, the L+P group showed an approximate reduction in miR-33 expression of 0.31 ± 0.02- and 0.20 ± 0.03-fold in PMVECs and mouse lungs (P < 0.05 or 0.001, n = 3 or 5), respectively, and these reductions were statistically significant compared with miR-33 expression in the LPS group (P < 0.05 or 0.001, n = 3 or 5).

### 3.4. RIP140 expression in PMVECs and mouse lungs

**Figure 4:**
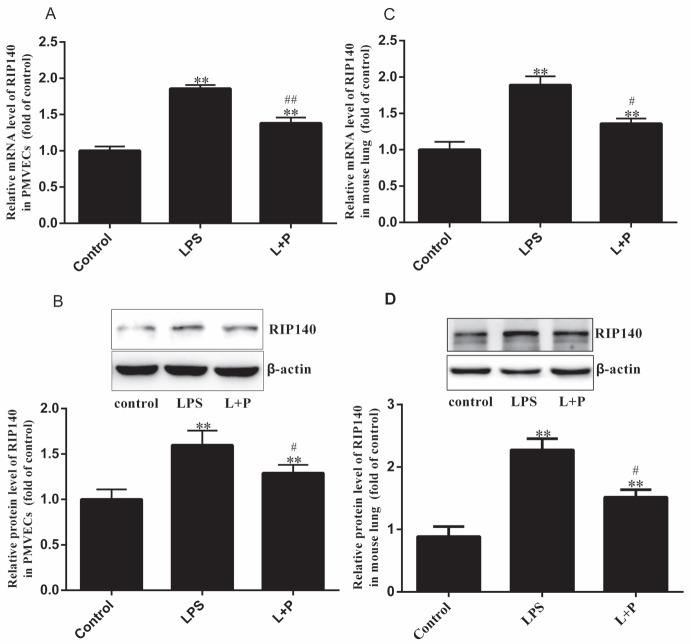
RIP140 expression in PMVECs and mouse lungs treated with LPS. RIP140 mRNA and protein levels in PMVECs (A and B) and mouse lungs (C and D) as analyzed by real-time quantitative PCR and Western blot analysis, respectively. Values are presented as the mean±SD (PMVEC n=3; mouse n=5). ** P< 0.001, the LPS group compared with the control group; #P < 0.05 and ##P < 0.001, the L+P group compared with the LPS group.

Given that RIP140 is the target gene of miR-33 (10), we assessed whether PMVECs and mouse lungs express RIP140 and assessed the effect of inflammation on RIP140 expression in PMVECs and the lungs of ALI mice. First, we measured RIP140 expression by qPCR and Western blot analysis. Figure 4 reveals that RIP140 is expressed in PMVECs and mouse lung tissue. RIP140 mRNA and protein levels were approximately 1.86 ± 0.11- and 1.6 ± 0.17-fold (Figures 4A and 4B, P < 0.001, n = 3) increased, respectively, in PMVECs after LPS treatment compared with control PMVECs. We also added 100 μmol/L PDTC before LPS treatment, which attenuated PMVEC lesions, decreased the release of LDH into the culture medium (Figure 2A), and suppressed the alterations in RIP140 expression induced by LPS (Figures 4A and 4B, P < 0.05 or 0.001, n = 3). Furthermore, we measured RIP140 expression in mouse lungs (Figure 4B), which revealed significant overexpression of RIP140 mRNA and protein in the LPS group compared with the control group (Figures 4C and 4D, P < 0.001, n = 5). The levels of RIP140 mRNA and protein were increased by approximately 1.89 ± 0.08- and 2.27 ± 0.15-fold in the LPS group and control group, respectively. In the L+P group, RIP140 mRNA and protein expression was increased by only approximately 1.36 ± 0.14- and 1.52 ± 0.13-fold (Figures 4C and 4D, P < 0.05, n = 5), respectively. These levels were reduced compared with those in the LPS group (P < 0.05, n = 5). Based on these results, we inferred that RIP140 expression was negatively correlated with miR-33 expression in PMVECs and mouse lungs.

## 4. Discussion

In this study, we discovered that miR-33 expression was reduced, whereas the target gene of miR-33, RIP140, was overexpressed in both LPS-treated PMVECs and BALB/c mice. However, PDTC relieved these alterations. Clearly, both miR-33 and RIP140 expression are tightly associated with inflammation.

Atherosclerosis is a chronic inflammatory process, and microRNA-33 is involved in atherosclerosis; therefore, miR-33 is also thought to be associated with inflammation (6,7,25,26). For example, inhibition of LPS-induced NF-κB activation further reduced miR-33 expression and enhanced ABCA1 expression and cholesterol efflux in macrophages (25,26). Moreover, transfection of Raw264.7 macrophages with miR-33 suppressed the LPS-stimulated production of TNF-α and IL-1β mRNA. Consistently, the levels of secreted TNF-α and IL-1β in cell culture media were also reduced (11). These studies only show that miR-33 regulates inflammation in macrophages but not in other cells and tissues. Moreover, the study by Guo et al. demonstrated that an inflammatory stimulus (LPS) can modulate the expression of miR-33 in the lungs according to miRNA microarray analysis but did not confirm this finding by other experimental techniques (8). However, the present results (Figure 3B) confirmed that the expression of miR-33 was downregulated in the lungs of ALI model mice. This finding suggested that miR-33 may be involved in inflammatory reactions of the lungs. Furthermore, when mice were intraperitoneally injected with LPS plus PDTC, which can attenuate the inflammatory response by inhibiting NF-**κB** (16,17,20), the changes induced by LPS were reduced compared with those in mice treated with LPS only. Clearly, these experimental phenomena further demonstrated that miR-33 expression depends on inflammation. In the process of ALI, PMVECs are the main component of the pulmonary alveolar-capillary membrane (4,5). Therefore, this study analyzed the possible function of miR-33 and RIP140 in PMVEC injury. Figure 3A demonstrates that miR-33 expression in injured PMVECs is significantly reduced (P < 0.001, n = 5) to levels similar to those noted in the lungs of ALI mice (Figure 3B). Altogether, these results indicate that miR-33 may play an important role in inflammatory injury in PMVECs and mouse lungs.

Previous studies have mainly demonstrated that RIP140 is expressed in metabolic tissues and negatively regulates energy homeostasis by affecting lipid storage and inhibiting the expression of genes involved in fatty acid oxidation and glucose metabolism (10–13). However, RIP140 in macrophages also acts as an NF-κB coactivator by recruiting CREB-binding protein (CBP) to modulate the TLR-induced production of proinflammatory cytokines, such as TNF-α and IL-1β (14). Therefore, changes in RIP140 expression can also regulate inflammation and affect TNF-α and IL-1β levels (14). For example, the overexpression or silencing of RIP140 in Raw264.7 macrophages enhances or reduces basal and LPS-stimulated TNF-α and IL-1β levels (27). In this study, we first demonstrated that both PMVECs and mouse lungs express RIP140 (Figure 4), and we found that RIP140 expression was increased in injured PMVECs and in LPS-induced ALI mouse lungs (Figure 4). However, RIP140 expression was reduced following PDTC treatment (Figure 4), as NF-κB activity was inhibited by PDTC (15). Therefore, RIP140 is clearly regulated by the degree of inflammatory injury in PMVECs and mouse lungs, and its expression is negatively associated with miR-33.

Although this study elucidated the relationship of miR-33 or RIP140 with ALI in vivo and in vitro, a major study limitation must be noted. Namely, this study did not alter the expression of miR-33 in PMVECs (or mice) to assess the detailed relationship of miR-33 and RIP140 with ALI. Altogether, our study demonstrated that 1) the expression of miR-33 and RIP140 participate in PMVEC injury in ALI and 2) RIP140 expression is negatively correlated with miR-33. These findings may help uncover the mechanism of ALI.

## Acknowledgments

This research was supported by grants from the National Natural Science Foundation of China (Grant No. 81560151 and Grant NO.81460468).
